# Defence Against Desiccation and Predation in Lophyohylini Casque-Headed Tree Frogs

**DOI:** 10.3390/toxins17060303

**Published:** 2025-06-16

**Authors:** César Alexandre, Pedro L. Mailho-Fontana, Bianca C. L. F. Távora, Marta M. Antoniazzi, Carlos Jared

**Affiliations:** Instituto Butantan, Sao Paulo 05503-000, Brazil

**Keywords:** Amphibia, Anura, casque-headed, Lophyohylini, defence, poison/venom, cutaneous glands

## Abstract

Casque-headed tree frogs (Lophyohylini) can have a very large and distinctive head characterised by hyperossification of their cranial skin. This type of skull was primarily associated with phragmosis, a behaviour in which the frog enters holes backwards and seals them with its head to prevent water loss in challenging environments. Further investigations revealed that hyperossification also gives rise to bony spines interspersed with skin poison glands. These peculiar anatomical features of the head make it challenging for predators to prey on the frogs in phragmosis. When bitten on the head, the bite pressure causes the spines to cross the poison glands, allowing the injection of toxins into the predator’s mouth. We studied the head morphology of different Lophyohylini species along with some characteristics of their cutaneous poison, both in the field and in the laboratory. These frogs exemplify distinct chemical defence strategies, highlighting the differences between venom and poison. Notably, some species can cause self-poisoning in predators by injecting poison (in this case, venom) through their head spines, similar to the use of fangs by snakes.

## 1. Introduction

Amphibians, unlike snakes, arachnids, and other venomous groups, lack a toxin injection system composed of venom-producing glands associated with teeth or stings and activated by muscles during attack or defence [1,2,3]. The class Amphibia possesses cutaneous poison glands distributed across the entire surface of their bodies, which are used against predators or microorganisms [3,4,5,6]. Amphibians, in general, are passive in relation to defence against predators. They can exhibit mimetic colour to avoid their detection. If detected, they often adopt stereotypical postures, escape, or display aposematic colours, which may confuse the predator or discourage potential attacks [7,8]. In general, the chemical defence system of amphibians depends on the bite of the predator. The predator itself, through the pressure of the bite, activates the amphibian’s cutaneous glands, causing poison to be deposited onto its oral mucosa [9]. In the case of toads (Bufonidae), which possess parotoid macroglands located just behind the eyes, the predator receives jets of poison in its oral mucosa [5]. Through this peculiar passive defence system [3], the predator is responsible for its own poisoning [3,5,9]. Unlike venomous animals, whose active defence aims to protect the individual, the passive defence of amphibians likely extends to the protection of the community: the attacked individual may die, but the predator/aggressor, when it survives the poison effects, is likely to “learn” to avoid similar prey [5,10,11].

In recent years, the distinction between venomous and poisonous animals (or between venom and poison) has increasingly become a target of several debates [3,12,13]. When facing an aggressor, tree frogs and salamanders, in addition to their typical passive chemical defence, are also able to actively inject their poison, which can be classified as venom in this case [3,14,15,16,17,18]. Among the tree frogs, this ability was primarily described in some species from the tribe Lophyohylini (Hylidae: Hylinae: Lophyohylini), widely known as casque-headed tree frogs. This group was well characterised phylogenetically only as recently as 2021 [19]. It consists of several species that can have broad, extensive skulls resembling flattened helmets, mainly covered by prominent bony spines interspersed with poison glands [16,18,19,20,21]. If these animals are attacked and bitten on the head, the pressure from the bite causes the spines to pierce the surrounding poison glands, thereby “injecting” toxins into the predator’s oral mucosa [16]. This unique apparatus displayed by these tree frogs contradicts the traditional classification, enabling their identification as both poisonous and venomous animals [3,16].

The peculiar morphology of casque-headed tree frogs’ skulls, which is often hyperossified and flat-shaped, resembling a lid, has been related to phragmosis [21]. This protective behaviour of using a body part to obstruct holes has been documented in ants, spiders, and a few tetrapods, such as armadillos [22,23]. In casque-headed tree frogs, the relationship between phragmosis and their skull shape was traditionally related to reducing water loss due to a decrease in the skin area exposed to the environment. Additionally, the mineralisation of the dermis of the cranial dermis (hyperossification) seems to make the skin more resistant to evaporation [23,24].

With an exclusively Neotropical distribution, they mainly inhabit South America, ranging from semiarid regions to humid forests the Lophyohylini are distributed across 10 genera, currently counting 93 species: *Corythomantis* (2 species), *Dryaderces* (2 species), *Itapotihyla* (monotypic), *Nyctimantis* (8 species), *Osteocephalus* (28 species), *Osteopilus* (8 species), *Phyllodytes* (16 species), *Phytotriades* (monotypic), *Tepuihyla* (9 species), and *Trachycephalus* (18 species) [19,25]. Three of these species have been the subject of recent toxinological studies: *Corythomantis greeningi*, from the Brazilian semiarid region [26]; *Nyctimantis brunoi*, from the coastal shrublands (restinga) of the Brazilian Atlantic Rainforest [27]; and *Nyctimantis siemersi*, found in flooded environments in open areas of Argentina [18].

In this study, we examined 11 species of Lophyohylini tree frogs, focusing on their natural history, behaviour, head morphology and defence mechanisms, including the analysis of the toxicity of their cutaneous secretions, as well as their protein profiles and enzymatic activities, aiming a comprehensive understanding of the strategies composing their antipredator defensive system. The tree frog *Boana albomarginata* (Hylidae: Hylinae: Cophomantini) was also studied in the same aspects as others, providing a basis for comparison [28]. In addition, we were able to sample two other species of tree frogs from Central America *Triprion spatulatus* and *Triprion spinosus* (Hylidae: Hylinae: Hylini), which have a modified skull but belong to another tribe [29].

## 2. Results

### 2.1. Natural History Features

During our fieldwork, we observed that more than half of the Lophyohylini species (Figure S1) collected inhabit areas of the Atlantic Rainforest biome (Table 1). We found some of the species in “Restinga” areas, which are coastal shrublands characterised by high solar incidence, salinity and strong winds (Table 1). Other species were collected in environments with even more challenging abiotic characteristics, distributed in the Brazilian semiarid regions (Caatinga), characterised by high solar incidence, very low average rainfall, and restricted seasonal precipitation (Table 1).

**Figure 1:**
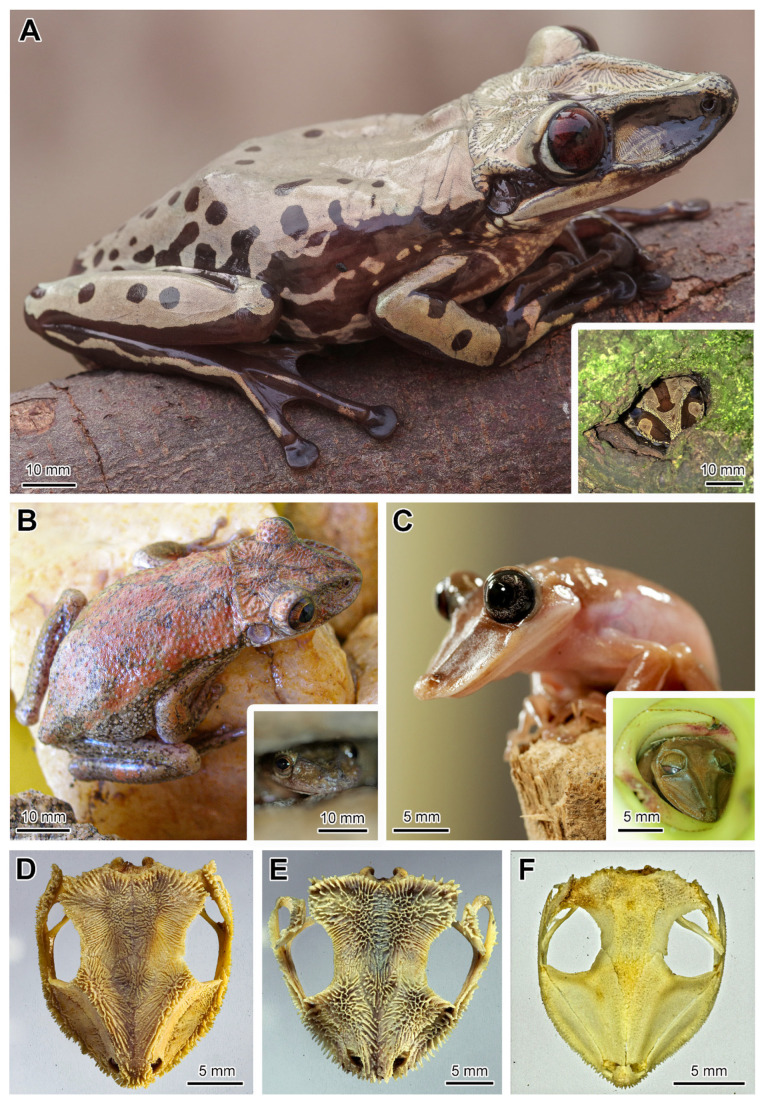
Representative species of casque-headed tree frogs (Lophyohylini) and their phragmotic behaviour in nature associated to anatomical aspects of the skull. (**A**) *Nyctimantis brunoi*. (**B**) *Corythomantis greeningi*. (**C**) *Nyctimantis arapapa*. Note the rough head of the different species due to the cranial spines that pierce the skin (see also Figure S2). The inserts from (**A**–**C**) illustrate the phragmotic behaviour in a tree hole, among rock crevices and in the central cup of a bromeliad, respectively. (**D**–**F**) Skulls of the three species highlighting the differences in shape and in the dermal spines. (**D**) *Nyctimantis brunoi*. (**E**) *Corythomantis greeningi*. (**F**) *Nyctimantis arapapa*.

Most of the casque-headed tree frogs here analysed, regardless of the biome in which they live, utilize natural shelters, such as tree holes, bromeliads, bamboo cavities, and rock crevices (Figure 1A–C, Figures S2 and S3 and Table 1). Usually, they enter the cavities backwards to perform phragmosis, exposing totally or partially the dorsal head to the environment (Figure 1A–C). Tree holes (Figure 1A) and bromeliads play a prominent role as shelter spots, as these plants retain rainwater between the basis of their leaves (axils) and in the central cup (phytotelma) (Figure 1C). Furthermore, in the semi-arid region (Caatinga), *Corythomantis greeningi* also utilises rock crevices as shelter (Figure 1B, Figures S3 and S4), which provide a more constant temperature and humidity, thereby maintaining a favourable environment even during drought periods. Phragmosis was not observed in *Boana albomarginata*, *Itapotihyla langsdorffii*, *Trachycephalus mesophaeus* and *Trachycephalus typhonius* (Table 1).

While inside the shelter, the phragmotic species typically maintain the head flexed towards the chest closing the entrance of the shelter, in a posture named chin-tucking behaviour (Figure 1A–C). When kindly disturbed with sticks, these species actively react by attacking the object with the head to protect themselves (Table 1 and Videos S1 and S2). While displaying this defensive behaviour inside the natural cavities, or even when manipulated, the frogs can exude great amounts of poison from the skin throughout the body, especially *Trachycephalus mesophaeus* and *Trachycephalus typhonius*.

### 2.2. Head Morphology

**Figure 2:**
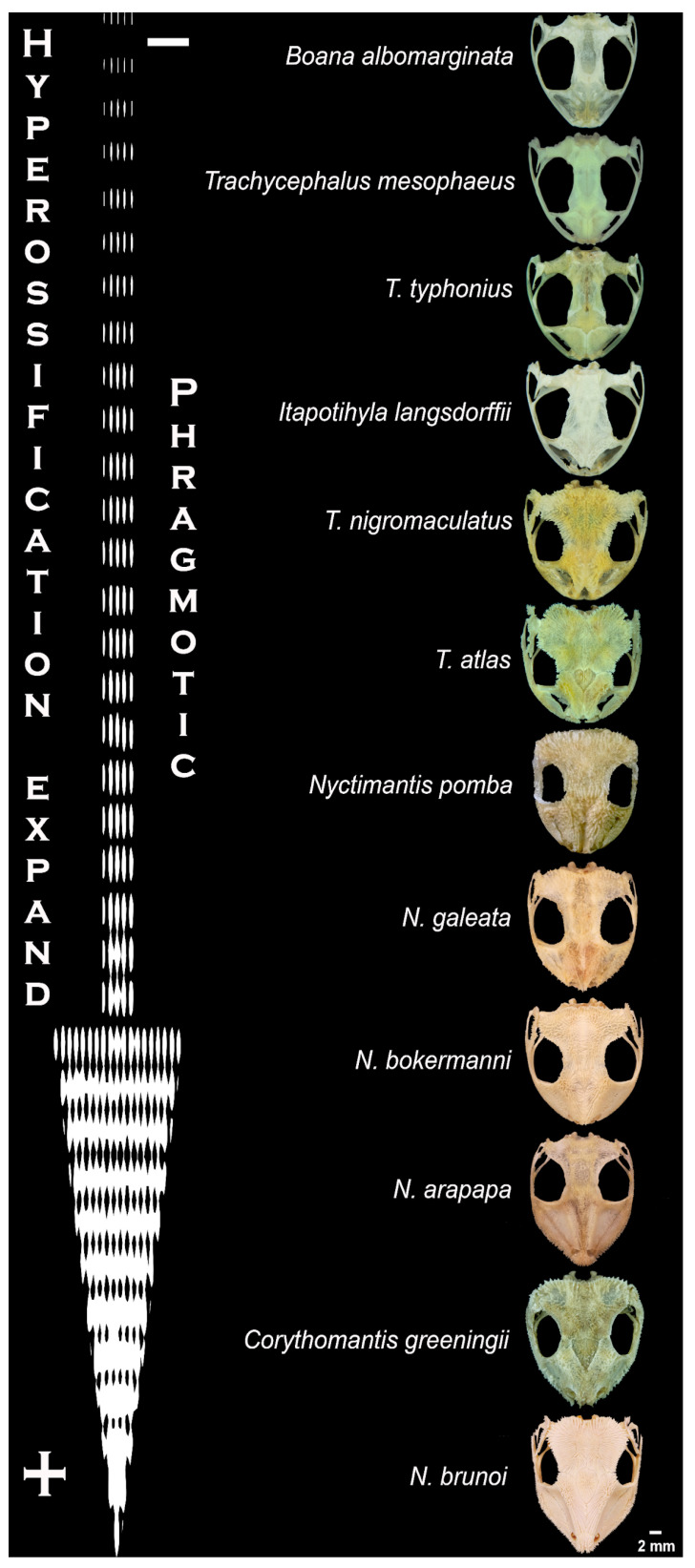
Skull bone extension and density of the casque-headed tree frogs (Lophyohylini) and the positive correlation with phragmosis.

Close observation of the head in different species under a stereomicroscope revealed exposed spines piercing the skin (Figure 1A–C), with exception of *Itapotihyla langsdorffii*, *Trachycephalus mesophaeus*, *Trachycephalus typhonius* and *Boana albomarginata*. The analysis of the anatomical features of all the skulls reveals that there is a clear difference between the phragmotic frogs in terms of bone area and bone density (i.e., grades of hyperossification) (Figure 2).

**Figure 3:**
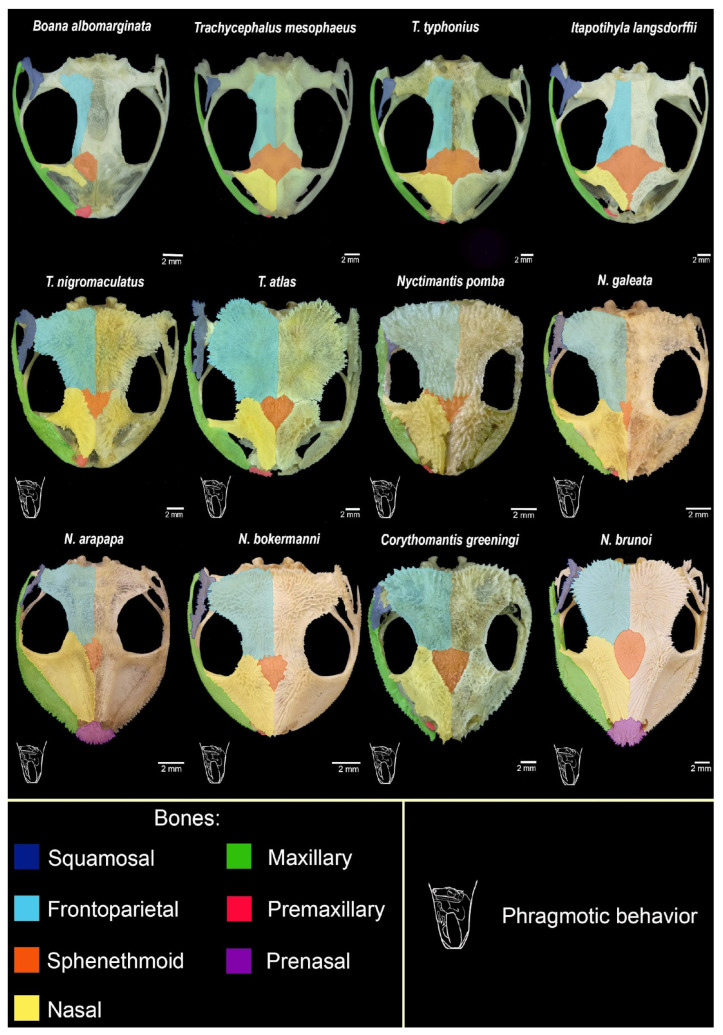
Differences in bone spines proportions in the skull of species studied. The colours represent the same bones in different species.

The surface of the skulls showed that most of the species have spines arranged side by side (Figure 1D–F and Figure 3). In the snout and particularly in the head posterior region, where they are disposed of as a crown, the spines are more prominent (Figure 3).

**Figure 4:**
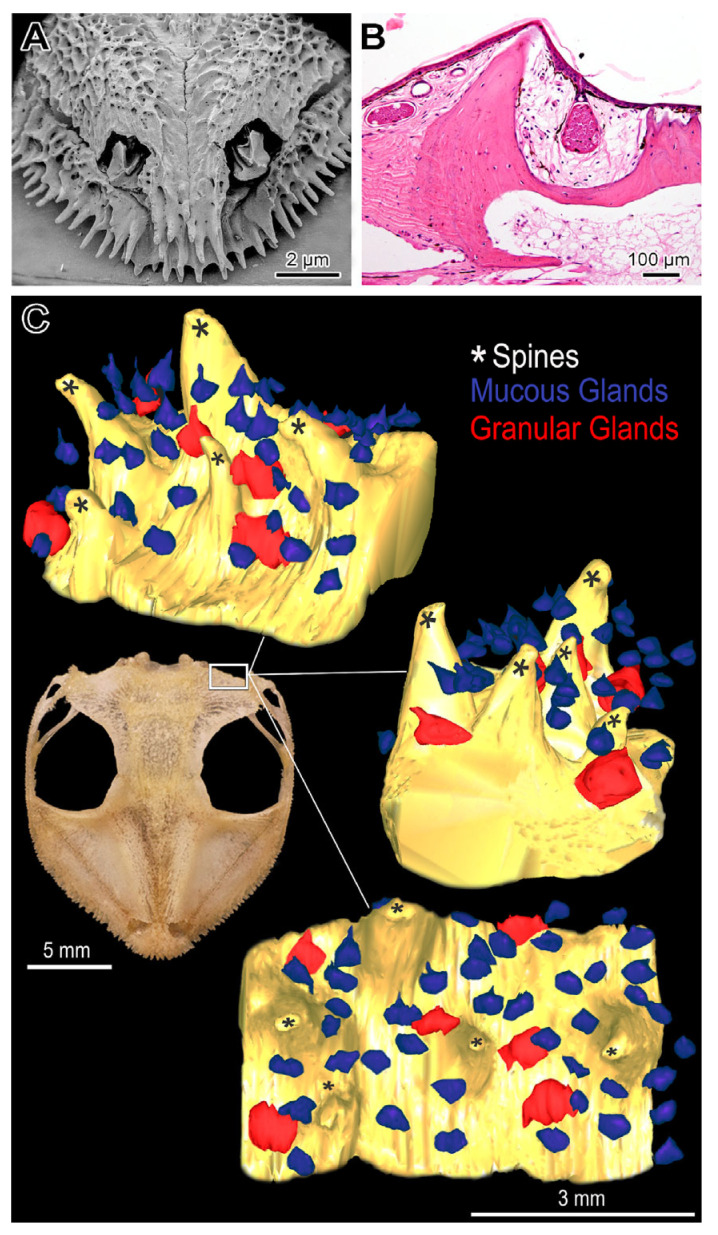
Representative scheme of the association between cranial spines and cutaneous glands in casque-headed tree frogs (Lophyohylini). (**A**) Scanning electron microscopy of the skull of *Corythomantis greeningi*. Note the prominent spines in the snout. (**B**) Histological section of the skull of *Nyctimantis arapapa* evidencing the cutaneous glands among cranial spines. (**C**) Three-dimensional reconstruction of histological sections from a fragment of *Nyctimantis arapapa* crown, revealing the skull spines and the cutaneous glands.

Except for *Itapotihyla langsdorffii*, *Trachycephalus mesophaeus*, *Trachycephalus typhonius* and *Boana albomarginata*, histological examination of the head demonstrated a clear association between the dermal bony spines and the cutaneous glands (Figure 4). The head of two species of *Tripion* analysed by histology showed similar morphological characteristics of Lophyohylini species, presenting a clear relationship between cranial ossified spines and poison glands (Figures S5 and S6).

### 2.3. Biological Activity and Biochemistry of the Cutaneous Poison

**Figure 5:**
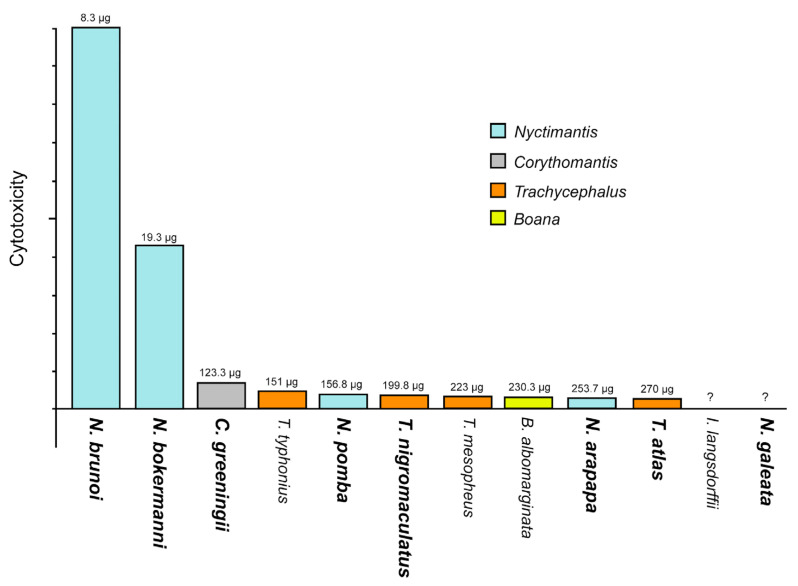
Comparison of cytotoxicity between the poisons of the casque-headed tree frogs. The bars represent the poison toxicity verified in cultured murine fibroblast cells (L929). The numbers above the bars represent the IC50 of each species. Note that the poison of *Nyctimantis brunoi* was the most toxic, followed by that of *Nyctimantis bokermanni*, which is 2.3 times less toxic than that of *N. brunoi*. The other poisons were 14 times (*Corythomantis greeningi*) to 32 times (*Trachycephalus atlas*) less toxic than that of *N. brunoi*. The poisons of *Itapothyla langsdorffii* and *Nyctimantis galeata* did not show cytotoxicity. The name of species highlighted in bold have a skull with spines. (?) Cytotoxicity could not be estimated at the doses used in the tests.

Among the cutaneous secretions of all species, *Nyctimantis brunoi* exhibited the highest cytotoxicity to fibroblast cells (IC50 8.34 μg/mL), corroborating previously observed in vivo lethality results [16]. The poison of *N. bokermanni* also demonstrated pronounced cytotoxicity, followed by *Corythomantis greeningi*, *Trachycephalus typhonius*, *N. pomba*, *T. nigromaculatus*, *T. mesophaeus*, *Boana albomarginata*, *N. arapapa* and *T. atlas*. Only the poison of *Itapotihyla langsdorffii* and *Nyctimantis galeata* did not show cytotoxic activity in all conditions tested (Figure 5).

**Figure 6:**
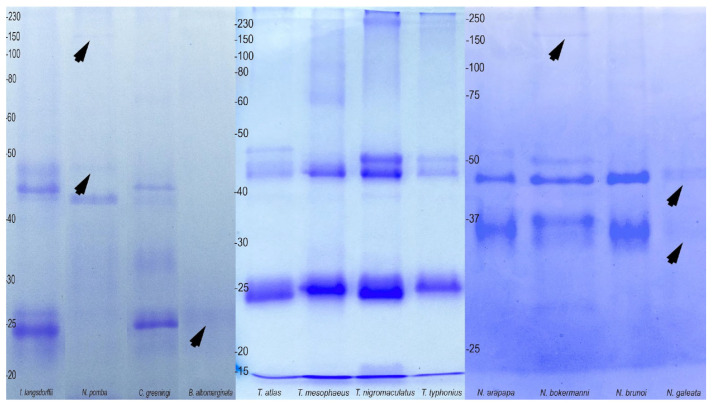
Protein profile in polyacrylamide gel (SDS-PAGE 15%) obtained from the skin secretion of the studied species. The arrows indicate low resolution bands.

Regarding poison composition, the protein profiles in SDS-PAGE gels were similar among species within each genus. In *Nyctimantis*, for example, two distinct groups of bands were observed (35 kDa and 45 kDa) (Figure 6). The poison of *Trachycephalus* exhibited a major band around 25 kDa, along with another group of bands encompassing masses between 40 and 50 kDa (Figure 6). The profile obtained from *Trachycephalus* was similar to that obtained from the poisons of *C. greeningi* and *I. langsdorffii* (Figure 6). *Boana albomarginata* presented a poor profile, with majority bands below 27 kDa (Figure 6).

**Figure 7:**
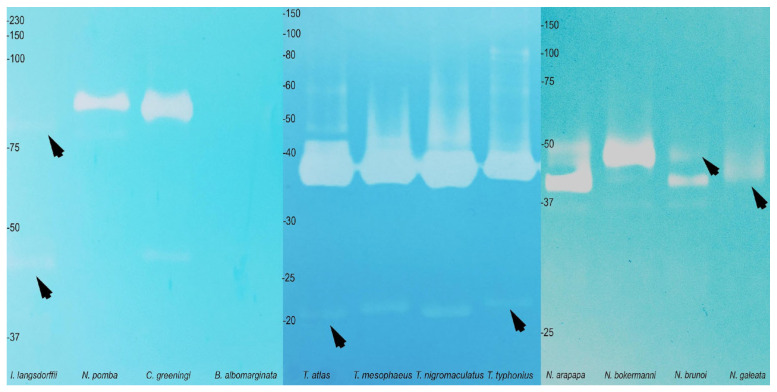
Hyaluronidase profile in polyacrylamide gel (SDS-PAGE 15%) obtained from the skin secretion of the studied species. The arrows indicate low resolution bands.

In terms of protease activity, zymographic tests performed on SDS-PAGE gels showed gelatinolytic, caseinolytic and fibrinogenolytic activities, particularly in the genus *Nyctimantis* (Figures S7–S9). The presence of hyaluronidases was observed in the secretions of all Lophyohylini species tested (Figure 7), regardless of the presence of cranial ossification. However, the hyaluronidase activity was particularly weak in *Itapotihyla langsdorffii* and *Nyctimantis galeata* and absent in *Boana albomarginata* (Figure 7).

## 3. Discussion

Due to the passivity of amphibians when confronted by predators, the front body, which is generally the first part of the animal to be bitten, has developed morphological adaptations strategically aimed at coping with predation. From a toxinological perspective, the development of cutaneous glands and macroglands—such as the parotoids in toads and other groups of anurans and urodeles [5,30]—is noteworthy. From a mechanical perspective, hyperossification is another important adaptation, creating a robust shield against bites. Hyperossification has evolved independently across different lineages at least 25 times [20]. In contrast, species that do not exhibit phragmosis tend to have limited cranial bone extension and less developed or absent spiny projections (Figure 3). Conversely, phragmosis seems to have initially developed as a strategy against desiccation. This strategy appears to have been successful, leading to its use in defence against predators, subsequently to development of spines.

One of the most interesting facts about antipredator defence among the Lophyohylini is the observation in many species of a combination of all these factors, where cranial ossification occurs in parallel with the presence of a significant number of poison glands, combined with phragmosis behaviour. Furthermore, the presence of hyaluronidase in the poisonous secretion seems to be a well-established phenomenon within the group, unlike other anurans in which this enzyme in their skin secretion has never been detected. The evolutionary tendency of this combination of factors becomes even stronger when one observes the clear correlation between the area and density of the cranial bone covering, the presence of cranial spines and the occurrence of phragmosis behaviour [20,26].

The unusual vertical head movement in some species of casque-headed tree frogs is entirely in line with the behaviour of phragmosis. Such movement is possible due to special tendons and muscles in the neck region, allowing the frogs to lower their heads toward their chests (chin-tucking behaviour) and facilitating better closure of the shelter entrance [21,31], which prevents the animal from being bothered or even captured by a predator. At the same time, phragmosis reduces moisture loss from inside the shelters [32].

Using the “chin-tucking behaviour”, at least the species *Corythomantis greeningi*, *Nyctimantis brunoi*, and *Trachycephalus nigromaculatus* have also developed an additional movement analogous to “goring”, investing the head against the attacker [16,26,33]. This movement may favour faster poison injection, forcing the spines to pierce the glands and delivering toxins to the aggressor. The “chin-tucking” behaviour is quite evident when holding a specimen in a closed hand. While collecting *Corythomantis greeningi* in the semiarid region of the Brazilian state of Rio Grande do Norte, one of the authors (CJ) experienced poisoning firsthand. As described in [16], during collection, which was carried out without gloves, the tiny amount of “venom” injected in the hand palm through goring was enough to cause 5 h of intense pain in the entire arm.

*Nyctimantis rugiceps* from Central America, as well as *N. pomba*, which inhabits the Brazilian Atlantic Rainforest, are also associated with phragmosis, using bamboo hollows as shelter [34,35]. *N. arapapa* appears to be the only *Nyctimantis* that has its entire life cycle restricted to bromeliads, which serve as both shelter and breeding sites [36]. The other species in this genus, such as *N. bokermanni*, *N. brunoi*, and *N. galeata*, reproduce directly in puddles despite using bromeliads for shelter. Moreover, during this study, we corroborated previous observations indicating that *Trachycephalus* with cranial ossification appears to be associated with xeric environments [37].

The behaviour of blocking the entrances to holes and crevices in rocks with a highly ossified head provides the animal with significant protection against water loss. The casque-headed frog *Nyctimantis brunoi* demonstrated that phragmotic behaviour can result in water savings of over 90% [24]. However, at least in *Corythomantis greeningi*, phragmosis in openings does not appear to be very effective against desiccation [32]. This effectiveness may depend on a combination of factors that greatly vary among the Lophyohylini. Considering morphological adaptations, the most significant factors likely include the density of the cranial ossification in the upper part of the head (where the dermis transforms into bone), the extent of cranial bone extension and density, and the thickness of the head skin [38,39,40].

The biochemical results are likely to reflect the adaptations of casque-headed tree frogs that have evolved in different habitats or suggest distinct biochemical specialisations related to defence against predators. In the genus *Trachycephalus*, for example, the toxic potential is not correlated with the presence of cranial bony spines. It calls attention to the fact that in some species, the poison is surprisingly potent. For example, compared to the venom of snakes from the genus *Bothrops*, which are responsible for the highest number of snakebites in Brazil, the poison of *N. brunoi* is 25-fold more potent [16]. Moreover, together with toxicity, the apparent exclusive presence of hyaluronidase in Lophyohylini among all anurans (and perhaps amphibians), regardless of the presence of cranial ossification, demonstrates the potential of these tree frogs to cause damage to tissues. Such characteristics may facilitate the diffusion through the predator’s body of the other toxic molecules, similar to the mechanism already known for the venom of animals with local effects, such as some snakes, lizards, arachnids, wasps, and even octopuses [41,42]. This supposition is reinforced by the finding of a weak hyaluronidase activity in the species *Itapotihyla langsdorffii* and *Nyctimantis galeata* that did not show cytotoxic activity.

Our results reaffirm the toxic potential of Lophyohylini for chemical defence, which, in the case of these anurans, could be classified, regarding the peculiar casque head, as active and endowed with a poison injection system, although more rudimentary compared to the well-known system present in venomous animals. The concurrent use of poison (throughout the body) and venom (through the head) by the casque-headed tree frogs suggests a unique attack and defence system (ADS)—composed of an extensive and robust skull with dermal bony spines and cutaneous glands secreting a cocktail of potent toxins, including hyaluronidases. Moreover, ADS is complemented by special movements of the head and phragmotic behaviour. However, this system does not appear to be exclusive to this group. It is also observed in species from other groups of tree frogs, such as the Hylini of the genus *Triprion* (*T. spatulatus*, *T. petasatus*, and *T. spinosus*) from Mexico and Guatemala, which have been little studied from a toxinological perspective. Thus, it is likely that this system evolved independently in different groups of anurans. In these animals, the ADS shows a varied efficiency in envenomation, ranging from spineless species, such as *Itapotihyla langsdorffii*, *Trachycephalus mesophaeus*, *Boana albomarginata* and *T. typhonius*, to *Corythomantis greeningi* and *Nyctimantis brunoi*, which have largely ossified and spiny heads. The anterior region, represented by the snout, and the posterior region, represented by the crown, are probably the cranial areas with most significant activity within the ADS, as they have the highest concentration of spines. Among the species analysed, *C. greeningi*, despite being the third most lethal species, has the most effective ADS for envenomation, featuring larger and more prominent spines, along with a significant number of glands.

Although ADS is an essential factor, some species of casque-headed tree frogs that lack spines and do not perform phragmosis also exhibited cytotoxicity and hyaluronidase activity. Therefore, the presence of toxic secretions may have evolved as a characteristic of this group even before the development of spines. In any case, there is a direct association between the presence of spines and ossification in Lophyohylini, which reflects the extension and density of skull bones, characterising hyperossification. As a result, the most extensive and dense skulls are present in species with the highest phragmotic activity.

## 4. Conclusions

In addition to providing new data that expands our understanding of the behaviour and defence mechanisms of anurans, the Lophyohylini serve as an excellent example of the chemical defence strategies employed by amphibians, indirectly illustrating the distinction between venom and poison. While some species rely on completely passive defence, allowing their poison to act through absorption via the oral mucosa at a slower rate, others have developed the ability to actively inject venom, which triggers symptoms rapidly by reaching the bloodstream. Thus, the ‘venomous’ Lophyohylini completely contradicts the typical passive defence strategies of amphibians, including their ability to utilize active injection of toxins through deliberate goring movements. The movement forces the toxins to penetrate the aggressor’s body, functioning similarly to the muscles surrounding the glands in truly venomous animals. In this context, the spines act as piercing agents, fulfilling the same role as teeth or stingers. Finally, the presence of enzymes (hyaluronidases) helps the venom to reach the predator’s tissues and bloodstream rapidly.

## 5. Materials and Methods

### 5.1. Animals and Fieldwork

For this study, observations and collections of 11 species of Lophyohylini and one of Cophomantini (Hylidae) were carried out across a significant portion of their geographic distribution, covering approximately 4000 km along the Atlantic coast and inland Brazil. The collected Lophyohylini species included *Corythomantis greeningi*; *Itapotihyla langsdorffii*; *Nyctimantis arapapa*; *N. bokermanni*; *N. brunoi*; *N. galeata*; *N. pomba*; *Trachycephalus atlas*; *T. mesophaeus*; *T. nigromaculatus*; and *T. typhonius* (Figure S1). In an additional and speculative manner, for better support, we also included the Cophomantini (Hylidae) *Boana albomarginata* (Figure S1), and two species of Hylini (Hylidae), *Triprion spatulatus* and *Triprion spinosus*. Notably, the species *Corythomantis greeningi* and *Nyctimantis brunoi* have been observed and collected by our group on expeditions since 1989. During our recent expeditions, we have successfully observed and collected little-known species considered rare, such as *N. galeata* [43] and *N. bokermanni* [44], as well as species with very restricted distributions, such as *N. arapapa* [31], and even species threatened with extinction, such as *N. pomba* [34].

Four field trips were carried out, covering the second half of 2019 to March 2022. Different Brazilian phytophysiognomies were covered according to the occurrence of each species, encompassing five states and totalling just over 30 days of work.

Once found, the animals were gently stimulated with a twig to verify basic defensive behaviours. Immediately after collecting, two to five specimens of each species were euthanised by intraperitoneal injection of thiopental (50 mg/kg) in combination with lidocaine (10 mg/mL) to preserve the morphological characteristics closest to those of animals in nature. Immediately afterwards, the specimens were preserved in a buffered paraformaldehyde solution at pH 7.2 for 72 h. After collecting the tissues of interest, the specimens were kept in 70% alcohol in the Structural Biology Laboratory.

Additional specimens were kept in the Vivarium of the Structural Biology Laboratory at the Butantan Institute in 45 L plastic boxes containing dry branches as perches, plastic or rubber tubes for shelter, and water that was frequently replenish. The animals were fed twice a week with varying amounts of crickets (*Gryllus assimilis*) and cockroaches (*Nauphoeta cinerea*).

The study was conducted with prior approval from Brazilian Chico Mendes Institute for Biodiversity Conservation (ICMBio-SISBIO-65237-7). All experimental procedures were performed in accordance with the relevant international guidelines and regulations and were approved by the Ethics Committee on Animal Use of Instituto Butantan, CEUA 8465270919 (ID 001887), on 16 October 2019.

### 5.2. Cranial Anatomy

Two individuals of each species (except *Nictymantis pomba*) were used to analyse cranial bone anatomy. Soft tissues were extracted using immersion in hydrogen peroxide (40 vol) in addition to sodium hypochlorite (3%) [45]. The corrosion time varied according to the needs of each sample, alternating immersion in the solutions and manual material removal. Cranial structures were identified and named according to classical taxonomy [46]. The term “cranial bone extension” adopted in this paper refers to the area and the bone volume occupied by the skull. The skull is commonly called “hyperossified” when composed of very voluminous bones [20]. The term “bony projections” refers to rounded or pointed protrusions or spines that emerge from the surface of the skull.

### 5.3. Histology

In all species analysed (except *Nictymantis pomba*), we histologically studied the posterior region of the skull, here called the “crown”, and the anterior region of the animals’ heads, the “snout”. We verified intraspecific variations and investigated the possible existence of patterns between the genera and aspects of Natural History. Fragments of the tegument in the dorsal region were also evaluated for comparisons between the skull and the back. Cranial decalcification was performed using a 4% EDTA solution for 30 to 65 days ([47], adapted). All samples were dehydrated in ethanol crescent series and embedded in plastic resin (Historesin, Leica Microsystems Nussloch GmbH, Heidelberg, Germany) or paraffin. Sections were obtained using a Leica 2255 microtome (Leica Microsystems, Nussloch GmbH, Heidelberg, Germany), with variable thickness between 0.5 and 6 μm.

In addition to the species collected during fieldwork, we examined the tree frogs *Triprion spatulatus* and *T. spinosus*, from Central America (provided by Prof. Edmund D. Brodie Jr. and Prof. Jonathan M. Campbell), which also have a spiny skull even though they do not belong to the Lophyohylini tribe. Cranial bone images of the two species come from the Morpho Source website, with *T. spatulatus* under ID B9848D79-6233-4503-8832-EB35AF95D9999 and *T. spinosus* under ID 87602/m4/M11850.

The histological sections were subjected to different staining techniques, using hematoxylin-eosin and toluidine blue-fuchsin to analyse basic morphology in paraffin and resin, respectively. To better understand the composition of the secretion of the different types of glands, different histochemical tests were performed: PAS—periodic acid + Schiff, to reveal sites rich in neutral polysaccharide; alcian blue pH 2.5, to reveal sites rich in acidic polysaccharides; bromophenol blue, to reveal sites rich in proteins; and von Kossa, to reveal sites rich in calcium. The sections were photographed on an Olympus BX51 microscope coupled to an Olympus DP 73 digital camera, and the images were captured using CellSens Standard 1.16 software. To perform the three-dimensional reconstruction of the Attack and Defence System (ADS), a fragment of the periphery of the frontoparietal bone of *Nyctimantis arapapa*, which has a very extensive and hyperossified cranial region, was used. The reconstruction was performed from 119 serial sections using Reconstruct 1.1.0.0 software (copyright © 1996-2007, John C. Fiala) [48], resulting in a reconstruction equivalent to 5.95 mm^2^ of the region.

### 5.4. Collection of Cutaneous Secretion

The species’ secretions were collected by immersing and massaging the specimens in deionised water or PBS 7.4 inside a sterile plastic bag [49]. This method causes mechanical stimulation of the cutaneous glands by gently compressing of different regions of the animals’ bodies.

After extraction, the material was filtered through a “Cell-Strainer 40 μm” filter and separated into aliquots. The protein concentration of the poison was determined using the “NanoVue Plus Spectrophotometer GE Healthcare” optical density reader. To minimize the chance of loss of poison activity, cytotoxicity and zymography experiments were performed immediately after collection, with other aliquots kept in the refrigerator and others in a freezer at −20 °C.

### 5.5. Cytotoxic Activity

Cytotoxicity tests were performed using a cell line of normal mouse fibroblasts (L929) maintained and tested in culture. The Effective Concentration (EC50) was calculated, which refers to the concentration that kills 50% of a cell population. The cutaneous secretion of the frogs for this test was extracted directly into PBS 7.4.

The cells, kept in liquid nitrogen, underwent a thawing process and were placed in 25 cm^2^ cell culture bottles containing RPMI-1640 medium supplemented with 10% foetal bovine serum, 2 mM glutamine, 10,000 U/mL penicillin and 10,000 μg/mL streptomycin. The cell line was kept in an incubator at 37 °C, with a humid atmosphere and 5% CO_2_, until confluence.

For the treatment of cell lines with crude cutaneous secretions from tree frogs, L929 cells were plated at a concentration of 3 × 105 cells/mL (3 × 104 cells/well) of complete RPMI-1640 medium in 96-well cell culture plates. After cell adhesion, the fractions were serially diluted at a ratio of 2 with RPMI-1640 medium and distributed in quadruplicate on the culture plate. The assays were performed with treatments of 6, 24 and 48 h of incubation at 37 °C in a humidified atmosphere with 5% CO_2_, with each sample of fresh venom, and were repeated three times. As a negative control, wells with cell cultures free from treatment with cutaneous secretion were maintained. After treatment, cell viability was assessed using the MTT assay [3-(4,5-dimethy-2-lthiazolyl)-2,5-diphenyl-2H-tetrazolium bromide [50]. Readings were performed in an ELISA reader (Multiskan EX^®^) at a wavelength of 540 nm.

### 5.6. Polyacrylamide Gel Electrophoresis in the Presence of Sodium Dodecyl Sulfate (SDS-PAGE)

To investigate the diversity of proteins in the secretions of the species studied, samples containing 8 μg of proteins were diluted in 15 μL of sample buffer and applied to a 15% polyacrylamide gel (PAGE) containing sodium dodecyl sulphate (SDS) under non-reducing conditions [51]. After the proteins were separated by electrophoresis, the gels were stained with Coomassie Brilliant Blue.

### 5.7. Zymography

The presence of proteases in the secretions was evaluated by the zymography electrophoresis technique [52], using casein, gelatin, hyaluronic acid and fibrinogen as substrates in 15% SDS-PAGE gels. After the end of electrophoresis, the gels containing casein, gelatin and fibrinogen were processed and stained with Coomassie brilliant blue. Alcian blue was used as dye only for the hyaluronic acid gel. After staining, the gels were immersed in decolorising solutions. As a result, clear areas in the gel indicate the degradation of the substrate used and, therefore, the occurrence of enzymatic activity at a given molecular mass.

## Figures and Tables

**Table 1:** The studied casque-headed tree frogs and the main individual environmental characteristics.

Species	Biome	Microenvironment	Phragmosis	Head Attack	Cranial Spines
*Boana albomarginata*	Atlantic Rainforest *	Not specific	No	No	No
*Corythomantis greeeningii*	Caatinga	Rock crevices	Yes	Yes	Yes
*Itapotihyla langsdorffii*	Atlantic Rainforest *	Not specific	No	No	No
*Nyctimantis arapapa*	Atlantic Rainforest	Bromeliads	Yes	Yes	Yes
*N. bokermanni*	Atlantic Rainforest *	Bromeliads	Yes	Yes	Yes
*N. brunoi*	Atlantic Rainforest *	Bromeliads/tree holes	Yes	Yes	Yes
*N. galeata*	Caatinga	Bromeliads	Yes	Yes	Yes
*N. pomba*	Atlantic Rainforest	Bamboo	Yes	Yes	Yes
*Trachycephalus atlas*	Caatinga	Bromeliads/palm trees	Yes	Yes	Yes
*T. mesophaeus*	Atlantic Rainforest	Not specific	No	No	No
*T. nigromaculatus*	Atlantic Rainforest	Bromeliads/tree holes	Yes	Yes	Yes
*T. typhonius*	Savannah	Not specific	No	No	No

* Areas under the influence of marine salinity.

## Data Availability

The original contributions presented in this study are included in the article/Supplementary Materials. Further inquiries can be directed to the corresponding author.

## Supplementary Materials

The following supporting information can be downloaded at: https://www.mdpi.com/article/10.3390/toxins17060303/s1. Figure S1: Species studied; Figure S2: *Nyctimantis brunoi* in phragmosis in a bromeliad; Figure S3: *Corythomantis greeningi* in a rocky crevice during the reproductive station; Figure S4: *Corythomantis greeningi* lips; Figure S5: Toxin injection system in *Triprion spatulatus*; Figure S6: Toxin injection system in *Triprion spinosus*; Figure S7. Gelatinolytic profile in polyacrylamide gel (SDS-PAGE 15%) obtained from the skin secretion of the studied species; Figure S8. Caseinolytic profile in polyacrylamide gel (SDS-PAGE 15%) obtained from the skin secretion of the studied species; Figure S9. Fibronogeniolytic profile in polyacrylamide gel (SDS-PAGE 15%) obtained from the skin secretion of the studied species; Video S1: Defensive behaviour associated to phragmosis in *Nyctimantis brunoi*; Video S2: Defensive behaviour associated to phragmosis in *Trachycephalus nigromaculatus*. References [53,54,55,56,57,58,59,60,61,62,63,64,65,66,67,68,69,70,71,72,73,74,75,76] are cited in the Supplementary Materials.

## Abbreviations

The following abbreviations are used in this manuscript:

ADS	Attack and Defence System
SDS-PAGE J	Polyacrylamide gel electrophoresis in the presence of sodium dodecyl sulfate
