# Supplementary material for: Defence Against Desiccation and Predation in Lophyohylini Casque-Headed Tree Frogs
Source: Toxins (Basel). 2025 Jun 16;17(6):303. doi: 10.3390/toxins17060303 (PMC12197542; doi:10.3390/toxins17060303)
Supplement: Supplementary file 1 [file toxins-17-00303-s001.zip › toxins-3653450-supplementary.pdf]

Supplementary Materials: Defence Against Desiccation and Predation in Lophyohylini Casque-Headed Tree Frogs

César Alexandre, Pedro L. Mailho-Fontana, Bianca C. L. F. Távora, Marta M. Antoniazzi and Carlos Jared

The document file includes:

Species sampled and their locations
Figs. S1 to S9
Caption for Movie S1 and S2

Species sampled and their locations

Corythomantis greeningi

Among all species analysed, this is one of the most intriguing in terms of distribution and occupation, inhabiting xeric environments (semiarid and savanna) in the interior of Brazil [53–55].

Itapotihyla langsdorffii

Monotypic species, with a wide geographic distribution, ranging from Rio Grande do Sul to Sergipe states, but also with occurrences in neighbouring countries such as Argentina and Paraguay [56–58]. Individuals were observed during the nocturnal period in activity and at rest during the day among the branches of shrubs or lianas.

Nyctimantis arapapa

The species shows a highly restricted population and endemism and is known only from the south-eastern region of Bahia [31,59]. Since it is a strictly bromeliad species, depending exclusively on bromeliads to complete its entire life cycle [36], we sought to directly inspect the phytotelms.

Nyctimantis bokermanni

The species is known only for description information collected by [60]. It is distributed exclusively along the Brazilian south coastal region, from Santa Catarina to São Paulo states [61,44].

Nyctimantis brunoi

Among the species of this genus, it has the most extensive set of published information. It is endemic to the Atlantic Rainforest and distributed throughout a large part of its coastal extension, from São Paulo to Bahia states [27,62].

Nyctimantis galeata

This species has a very restricted distribution in the semiarid, with populations known only in one area of Bahia state, in the highlands of the municipality of Morro do Chapéu [63]. It can be found in specific environments, such as rocky outcrops, close to streams with bromeliads and shrubby vegetation on their banks, where they take shelter [43,64].

Nyctimantis pomba

The International Union for Conservation of Nature (IUCN) officially considers this species as Critically Endangered (CR). It is restricted to a single area on the banks of the Pomba River in the state of Minas Gerais [34], associated with bamboo areas, sheltering in holes that appear in their stalks [65].

Trachycephalus atlas

Officially, the species is restricted to the semiarid of several states of Northeastern Brazil [66–68].

Trachycephalus mesophaeus

The species is widely distributed throughout the Atlantic Rainforest region, from Rio Grande do Sul to Alagoas states [69]. It mainly spreads along forest edges but is frequent in coastal shrublands, occasionally even in anthropized areas [69,70].

Trachycephalus nigromaculatus

This species, typical from the Atlantic Rainforest, occurs from São Paulo to Bahia [71].

Trachycephalus typhonius

Among the species analysed in this study, it has the most extensive geographic distribution, occupying southern Central America and a large part of South America [72]. In Brazil, it is present in almost all biomes, covering the Amazon and Atlantic rainforests and savanna, extending to the borders of the semiarid region [72–76].

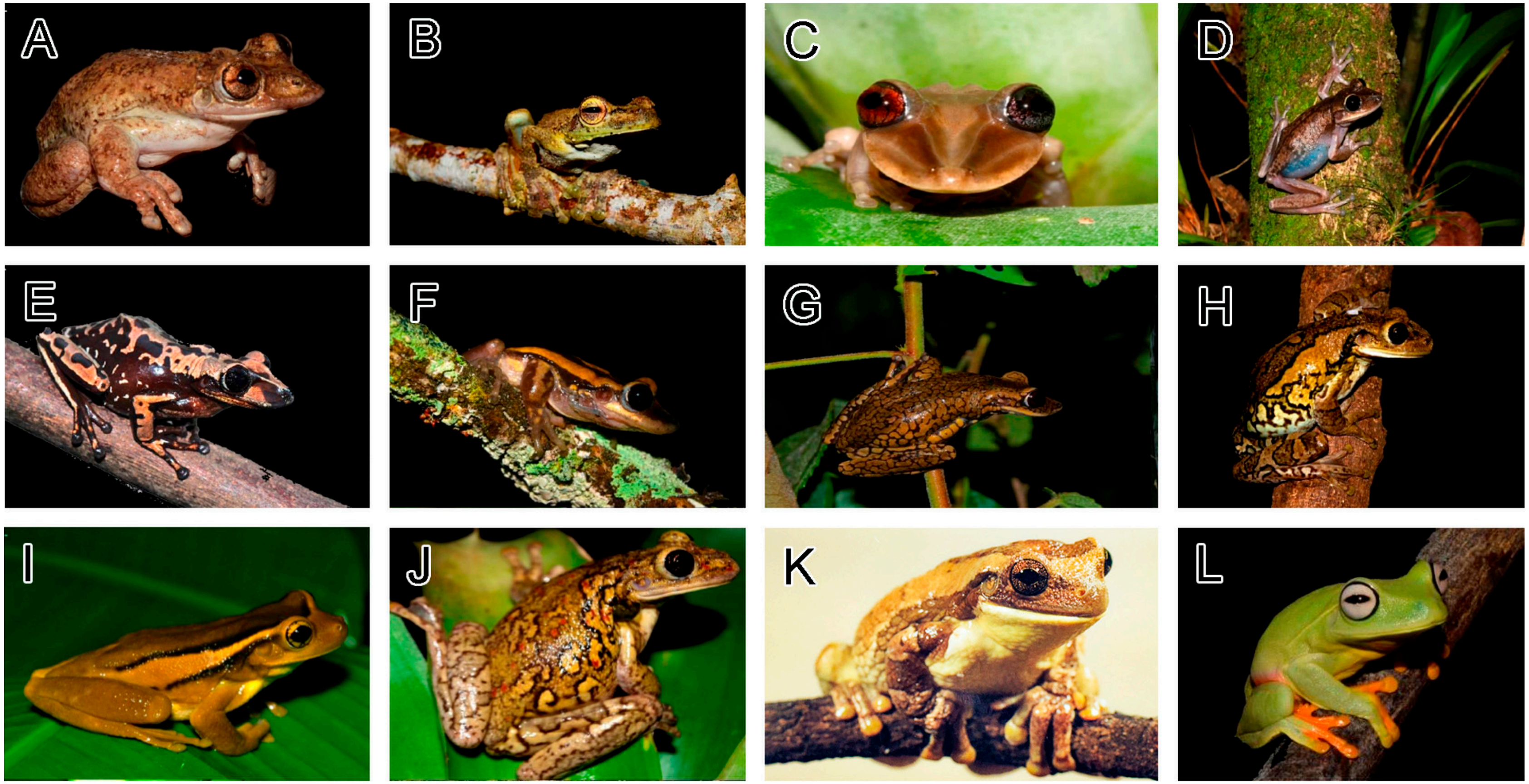

Figure S1. Species studied. (A) Corythomantis greeningi. (B) Itapotihyla langsdorffii. (C) Nyctimantis arapapa. (D) N. bokermanni. (E) N. brunoi. (F) N. galeata. (G) N. pomba. (H) Trachycephalus atlas. (I) T. mesophaeus. (J) T. nigromaculatus. (K) T. typhonius. (L) Boana albomarginata.

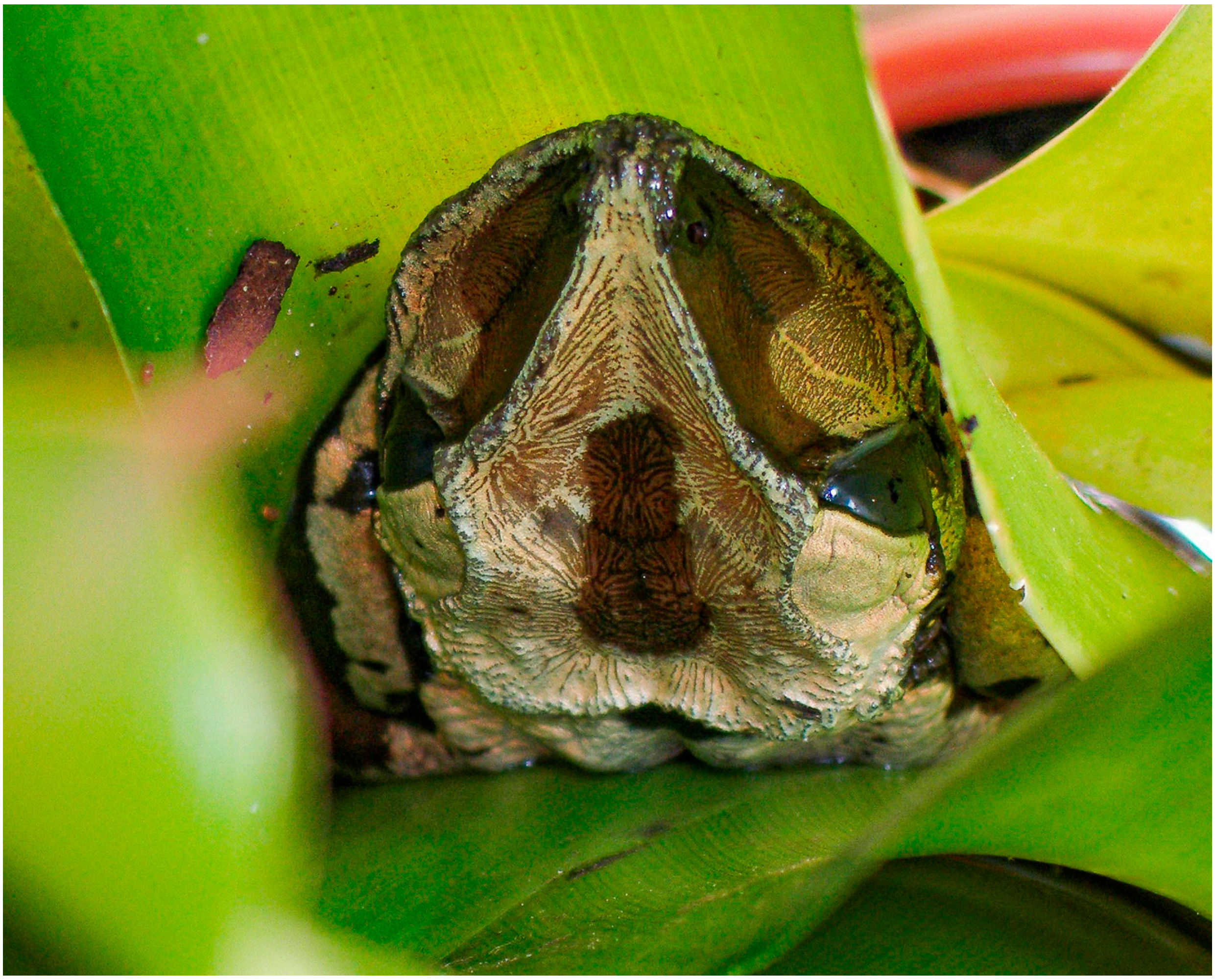

Figure S2. *Nyctimantis brunoi* in phragmosis in a bromeliad. Note the spines (darker lines) piercing the cranial skin.

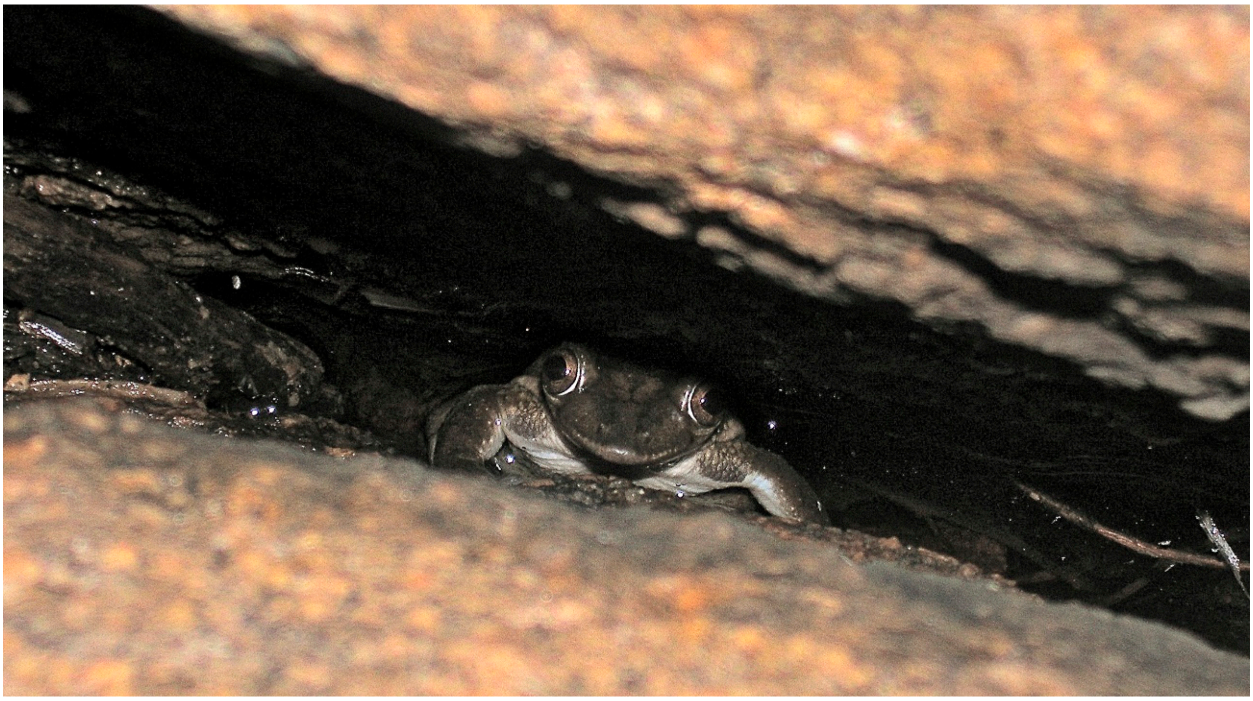

Figure S3. *Corythomantis greeningi* in a rocky crevice during the reproductive station.

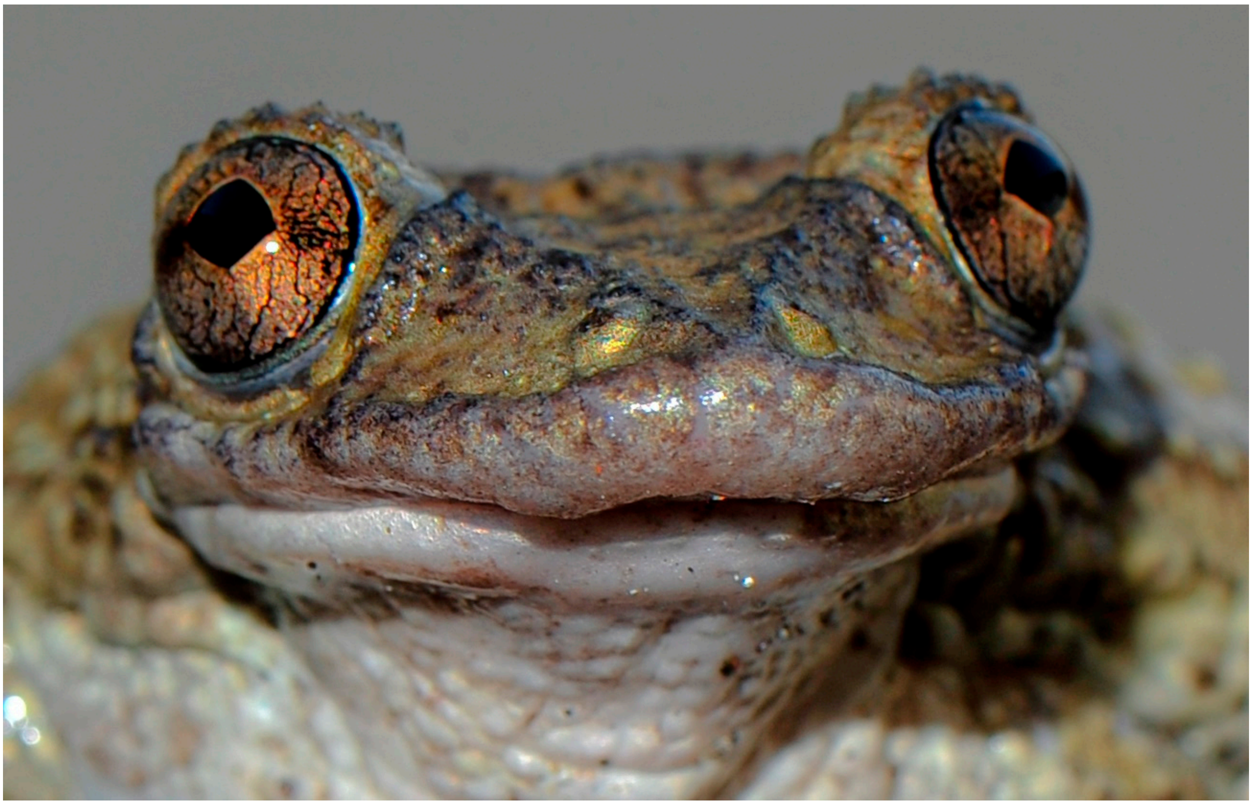

Figure S4. *Corythomantis greeningi* lips. Note the spines (darker spots) piercing the cranial skin.

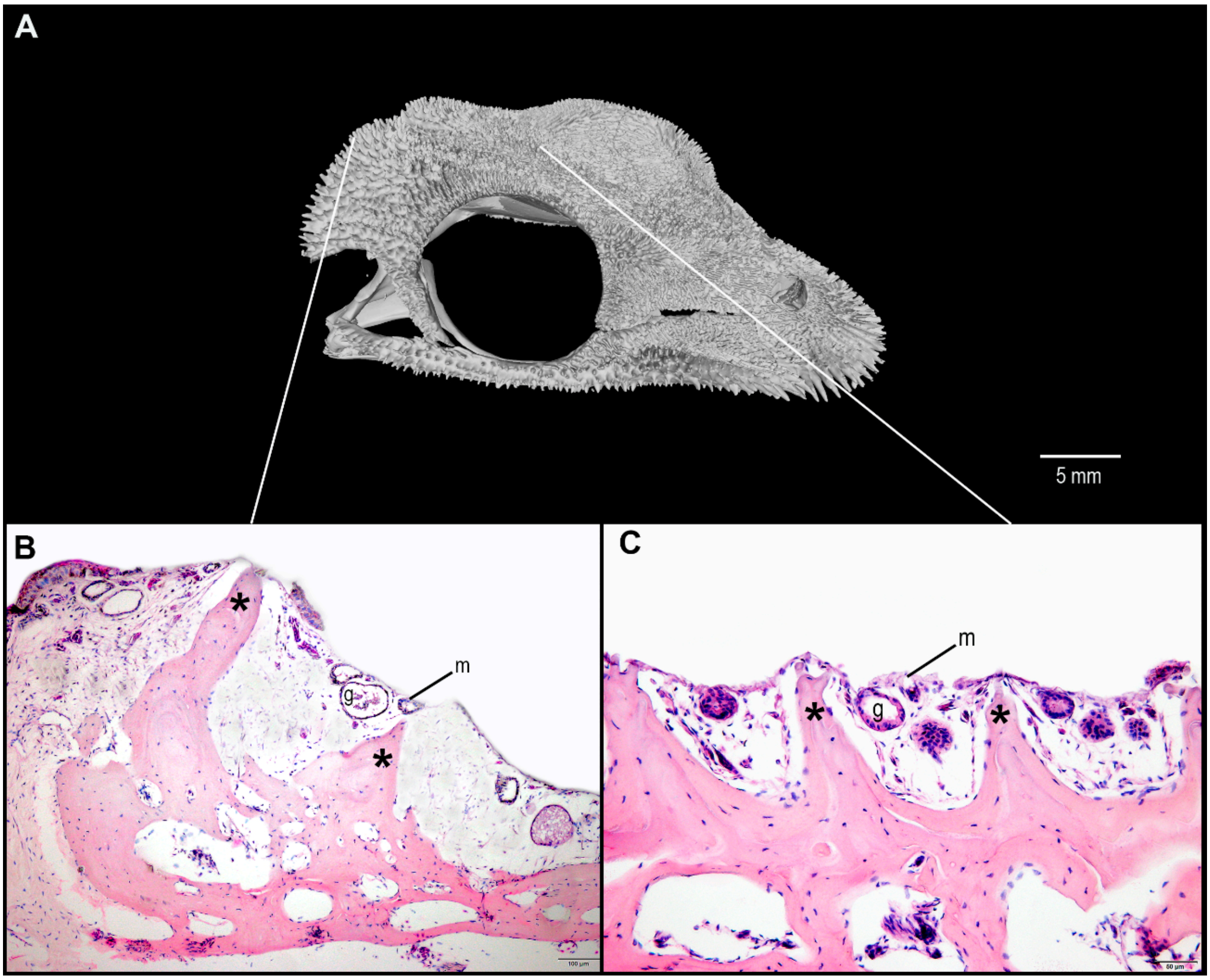

Figure S5. Toxin injection system in *Triprion spatulatus.* (A) Microtomography of the skull. (B) Association of granular glands (g) and mucous glands (m) with spines (*) in the posterior region of the skull. (C) Association of glands with spines in the central region of the skull. Microtomography image obtained from the open access Morpho Source website ID: B9848D79-6233-4503-8832-EB35AF95D9999.

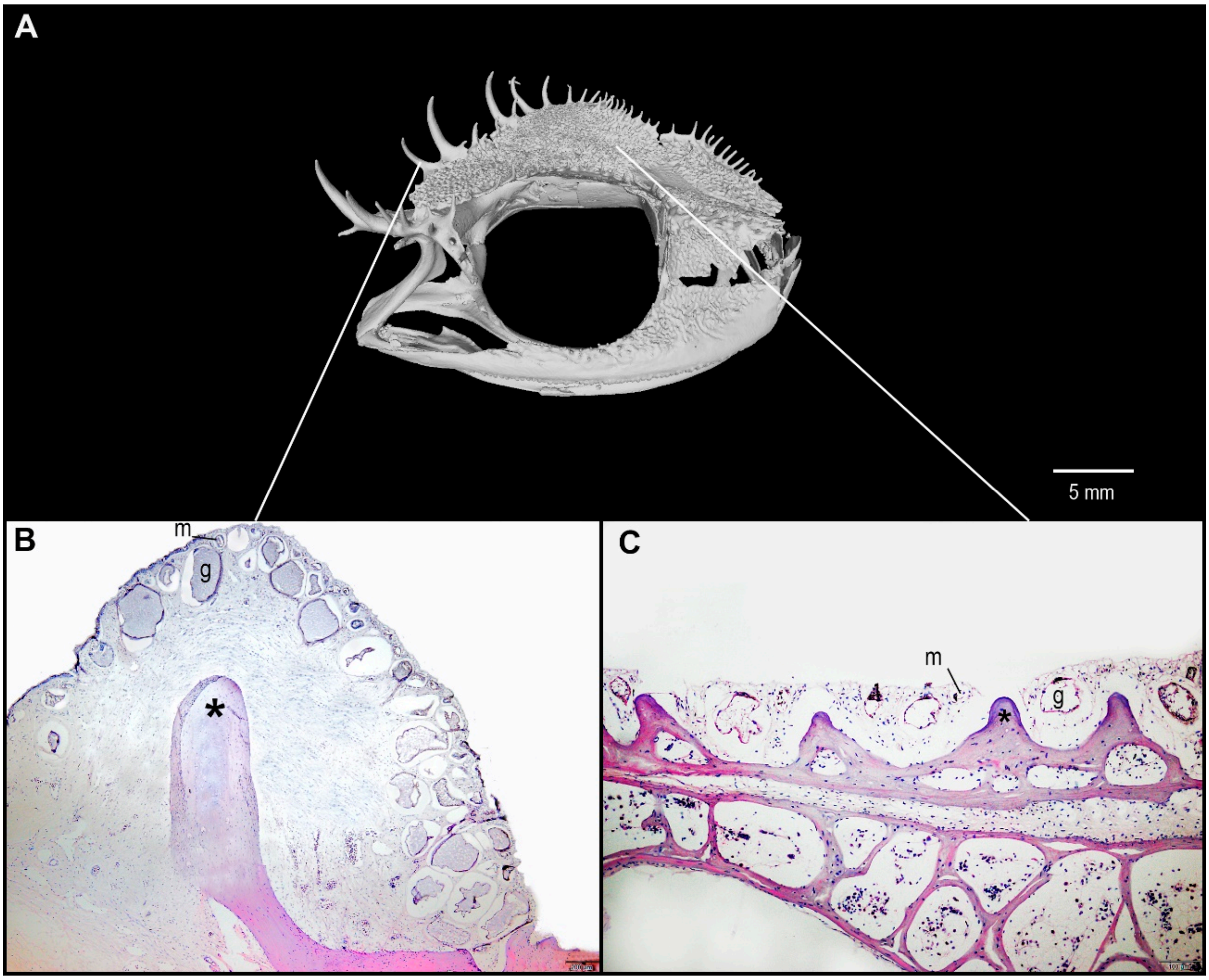

Figure S6. Toxin injection system in *Triprion spinosus.* (A) Microtomography of the skull. (B) Association of granular glands (g) and mucous (m) glands with spines in the posterior region of the skull. (C) Association of glands with spines in the central region of the skull. Microtomography image obtained from the open access website Morpho Source ID: 87602/m4/M11850.

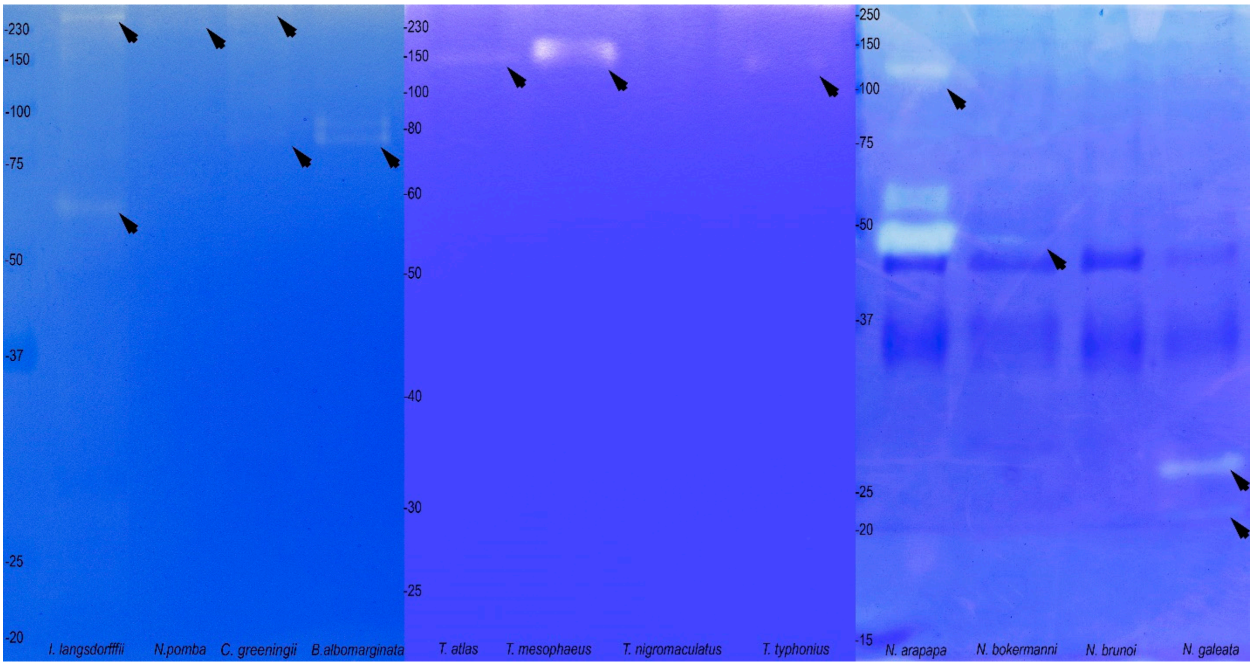

Figure S7. Gelatinolytic profile in polyacrylamide gel (SDS-PAGE 15%) obtained from the skin secretion of the studied species. The arrows indicate low resolution bands.

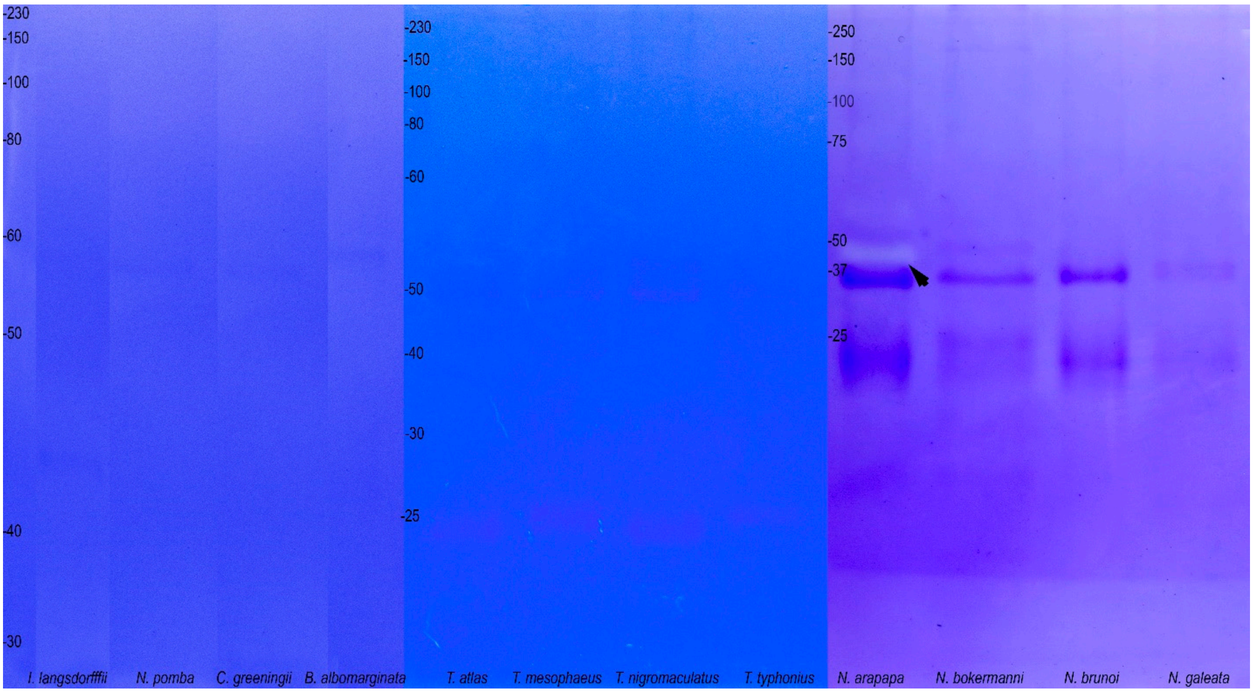

Figure S8. Caseinolytic profile in polyacrylamide gel (SDS-PAGE 15%) obtained from the skin secretion of the studied species. The arrows indicate low resolution bands.

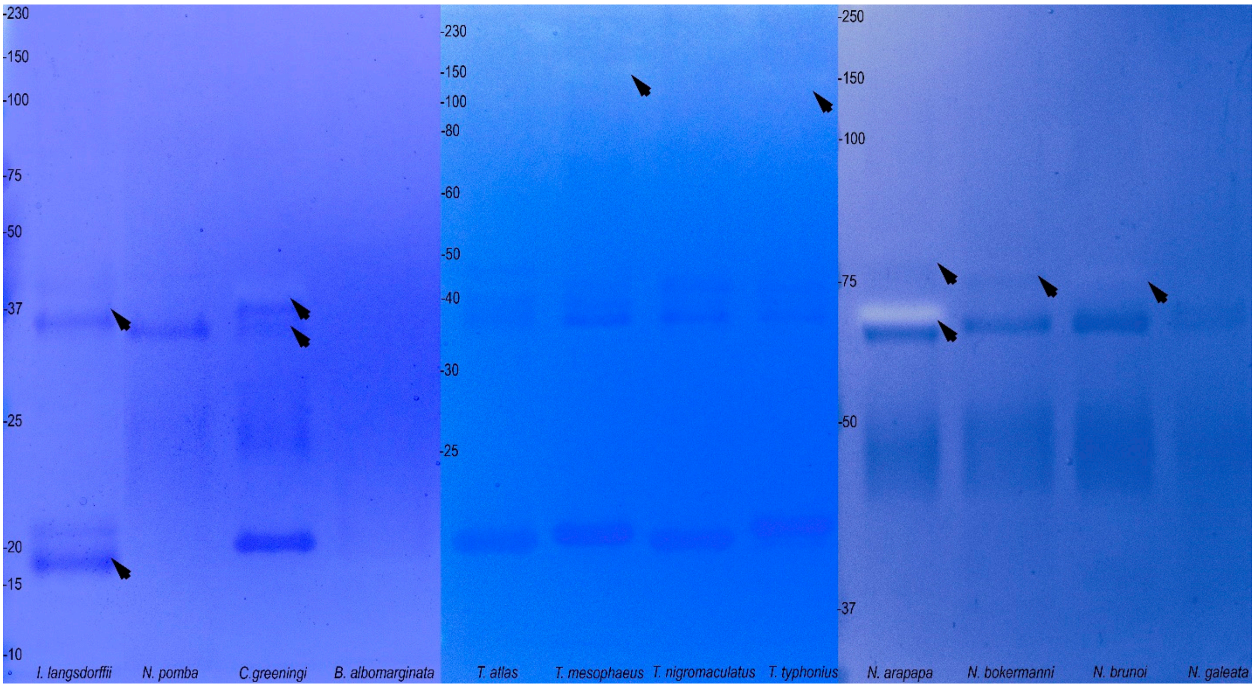

Figure S9. Fibronogeniolytic profile in polyacrylamide gel (SDS-PAGE 15%) obtained from the skin secretion of the studied species. The arrows indicate low resolution bands.

Movie S1. Defensive behavior associated to phragmosis in *Nyctimantis brunoi*. Access in: https://drive.google.com/file/d/14kbVYVympwK7aYQIvQeBpeiCz8TvoYsv/view?usp=drive_link

Movie S2. Defensive behaviour associated to phragmosis in *Trachycephalus nigromaculatus*. Access in: https://drive.google.com/file/d/1RAXNn_M6ek-W-0DmSTZWaVAmakIMBMs4/view?usp=sharing
